# Facile construction of mechanically robust and highly osteogenic materials for bone regeneration

**DOI:** 10.1016/j.mtbio.2025.101809

**Published:** 2025-05-03

**Authors:** Song Chen, Dachuan Liu, Qianping Guo, Li Dong, Huan Wang, Jiaxu Shi, Weicheng Chen, Caihong Zhu, Weishan Wang, Wei Xia, Miodrag J. Lukic, Helmut Cölfen, Bin Li

**Affiliations:** a Medical 3D Printing Center, Orthopedic Institute, Department of Orthopedic Surgery, The First Affiliated Hospital, MOE Key Laboratory of Geriatric Diseases and Immunology, School of Basic Medical Sciences, Suzhou Medical College, Soochow University, Suzhou, Jiangsu, 215000, PR China; b Collaborative Innovation Center of Hematology, Soochow University, Suzhou, Jiangsu, 215000, PR China; c Department of Orthopaedic Surgery, The First Affiliated Hospital, Shihezi University School of Medicine, Shihezi, Xinjiang, PR China; d Applied Materials Science, Department of Engineering Science, Uppsala University, Uppsala, Sweden; e Laboratory of Physics, “Vinca” Institute of Nuclear Sciences, National Institute of the Republic of Serbia, University of Belgrade, Mike Petrovica Alasa 12-14, Vinca, 11351, Belgrade, Serbia; f Physical Chemistry, Department of Chemistry, University of Konstanz, Universitätsstraße 10, 78457, Konstanz, Germany

**Keywords:** Bone regeneration, Biomineralization, Hydrogel, Load-bearing

## Abstract

Hydrogel-based materials exhibit great potential in tissue engineering. However, their mechanical weakness limits applications in hard tissue regeneration, especially under load-bearing conditions. Although various strengthening strategies have been applied, the achieved mechanical response of hydrogels still lags behind the mechanics of natural bone. In this study, we present a novel mineralization approach to fabricate mechanically robust and highly osteogenic mineralized hydrogels. Cross-linking between deprotonated chains of poly(acrylic acid) (PAA) and divalent cations has led to formation of hydrogels with a compressive strength and elastic modulus of 0.3 ± 0.1 kPa and 1.3 ± 0.2 kPa, respectively. Subsequent in situ formation of nano-calcium hydroxide crystals remarkably increased the compressive strength and modulus to 7.9 ± 0.6 MPa and 339.3 ± 31.4 MPa, respectively, surpassing those of trabecular bone. Moreover, the mineralized hydrogels demonstrated remarkable osteogenic potential in vivo, exhibiting immunoregulatory activity, promoting early angiogenesis, and accelerating fracture healing at weeks 4 and 8. The mechanism of osteogenesis was further revealed by transcriptome sequencing, indicating that the mineralized hydrogels regulated the translation of extracellular matrix and biomineralization. Overall, our study presents a pioneering and cost-effective method for fabricating materials with exceptional mechanical strength and strong osteogenic properties, offering a promising avenue for load-bearing bone repair applications of hydrogel-based materials.

## Graphical abstract

**Figure:**
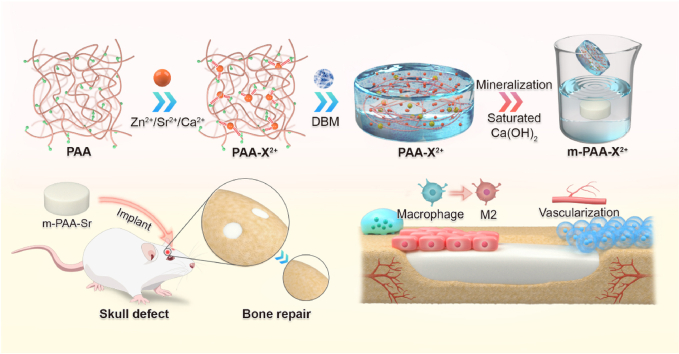

## 1 Introduction

Despite the variety of synthetic biomaterials developed during the past decades, autografts are still the golden standard in clinical practice for bone repair. However, the autograft harvesting process is accompanied with pain, risk of infections, and potential morbidity at the donor site. Therefore, bone substituting materials which provide sufficient mechanical support and promote the healing process are still highly needed [[1], [2], [3]].

Hydrogels are consisted of a three-dimensional network of crosslinked polymer chains and a large amount of water, and they are promising materials for tissue engineering [4,5], wearable electronics [6], and soft robotics [7]. The compressive strength of most hydrogels is in the range of 0.001–0.1 MPa and elastic moduli is < 1 MPa. Notably, widely employed tissue engineering scaffolds based on gelatin methacryloyl (GelMA) and silk fibroin methacryloyl (SilMA) hydrogels demonstrate even more restricted load-bearing capacities, typically showing compressive strengths between 2 and 50 kPa as documented in literature [8]. By incorporating hydroxyapatite nanofibers into GelMA matrices, the compressive strength of the hydrogel can reach up to 0.35 MPa [3]. Consequently, hydrogel-based biomaterials were mainly applied for non-load-bearing purposes (e.g., skin, muscles, cornea, and cardiovascular tissue) [[9], [10], [11]]. Various strategies have been applied to strengthen hydrogels and broaden their applications to hard tissue regeneration, i.e., bone and dental tissues: double network hydrogels [12,13], interpenetrating network hydrogels [14,15], fiber-reinforced hydrogels [16,17], sliding ring hydrogels [18], tetra-arm poly-(ethylene glycol) hydrogels [19], and nano-composite hydrogels [[20], [21], [22]]. However, most of high-strength hydrogels are unstable in aqueous media, limiting their applications under load bearing conditions [23]. Furthermore, preparing hydrogels with high strength usually involves complex synthetic chemistry and cytotoxic substances that are not suitable for biomedical applications. Therefore, the fabrication of mechanically robust, biocompatible and osteogenic hydrogel-based materials for bone substitution still remains a major challenge.

Recently, a so-called mineral plastic hydrogel based on physically crosslinking amorphous calcium carbonate (ACC) with polyacrylic acid chains has been developed [24]. It is shapeable, stretchable and self-healing, with the applications as inflammable foams [25], organic-inorganic adhesive [26] and mechanically adaptable ionic skin sensor [27]. These properties and possibilities make them prospective candidates for tissue repair. However, they are mechanically weak in aqueous media and their biological properties as implantable materials are elusive. A promising strategy to strengthen hydrogels and enhance their mechanical performance is forming inorganic phases uniformly within the hydrogel matrices [[28], [29], [30], [31], [32]]. This strategy is inspired by the composite nature of biominerals such as teeth and bone. Bone tissue mainly consists of collagen and carbonated hydroxyapatite. Collagen fibrils assemble into highly-oriented fibers, while hydroxyapatite crystals are uniformly distributed along the fibers. This composite nature and hierarchical structure contribute to excellent mechanical properties of bone tissue. Biomineralization-inspired strategies have been applied to prepare hydrogel-based composites with enhanced mechanical performance. For example, the compressive modulus of a hydrolyzed collagen-based hydrogel increased from 0.004 to 0.125 MPa after mineralization [33]. The interaction of calcium phosphate nanocrystals with polymer chains resulted in materials with improved mechanical properties, achieving a compressive strength and an elastic modulus of 1.7 and 2.1 MPa, respectively [34]. A recent study shows that the enzyme induced formation of amorphous calcium phosphate nanostructures within hydrogels resulted in tougher materials. The mineralized hydrogel possesses compressive strength of 1.3 MPa and elastic modules up to 300 MPa [35]. Via impregnation of hydrogels into the delignified wood followed by in situ mineralization of hydroxyapatite (HAp) nanocrystals, a hydrogel with compressive strength over 30 MPa were prepared [36]. These studies have showed that the mineralized hydrogels with improved mechanical strength possess great potential as scaffolds for hard tissue regeneration.

**Scheme 1:**
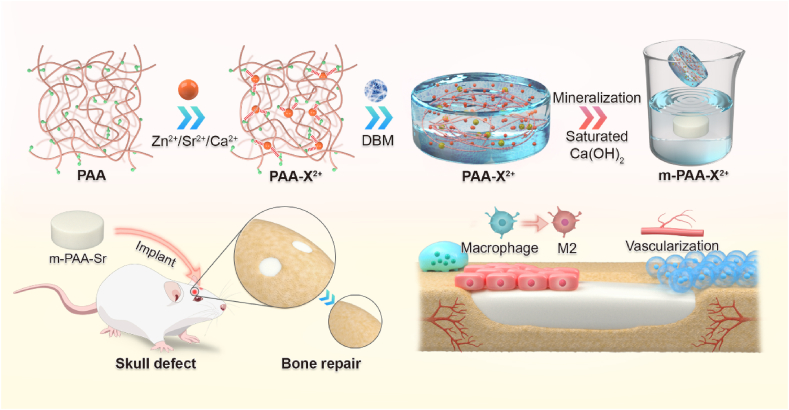
Illustration of the design and preparation of the mineralized hydrogels to repair a critical-size bone defect. The hydrogels with high mechanical strength were prepared by mineralizing the hydrogels crosslinked between deprotonated PAA chains and divalent cations. Blended with demineralized bone matrix, the strontium containing hydrogels showed good immunoregulatory activity and enhanced early angiogenesis and ultimately promoted fracture healing.

In addition to mechanical properties, osteogenic performance is crucial for the application of bone repair materials. Demineralized bone matrix (DBM), known for its osteoconductive and osteoinductive properties, has long been used in bone repair [37]. However, its limited mechanical strength restricts its broader application [38]. In this study, we introduce a novel mineralization approach to develop mechanically robust and highly osteogenic mineralized hydrogels (Scheme 1). These hydrogels were synthesized by interacting deprotonated polyacrylic acid chains with divalent metal ions and blending them with DBM. Subsequently, in situ mineralization was carried out using a Ca(OH)_2_ solution, resulting in hydrogels with mechanical properties comparable to those of natural trabecular bone. Significantly, these mineralized hydrogels demonstrated the ability to polarize macrophages towards the M2 phenotype, promoting early osteogenesis and ultimately facilitating skull regeneration. Transcriptome sequencing further elucidated the osteogenesis mechanism, revealing that the hydrogel-based materials modulate extracellular matrix translation and biomineralization processes.

## 2 Materials and methods

### 2.1 Chemicals

Poly(acrylic acid) (PAA, 25 %, Mw 240000) was purchased from Thermo Fisher Scientific. Anhydrous calcium chloride (CaCl_2_) and pepsin were obtained from Shanghai yuanye Bio-Technology Co., Ltd. Zinc chloride (ZnCl_2_) was obtained from Macklin. Sodium metasilicate pentahydrate (Na_2_SiO_3_**·**5H_2_O), strontium chloride hexahydrate (SrCl_2_·6H_2_O), calcium hydroxide (Ca(OH)_2_), and methanol were bought from Aladdin. Ethylenediaminetetraacetic acid disodium (EDTA·2Na) was bought from Beijing Solarbio Science & Technology Co., Ltd. Trichloromethane and HCl were supplied by Jiangsu Yonghua Chemical Co., Ltd.

### 2.2 Sample preparation

#### 2.2.1 Synthesis of PAA-Ca, PAA-Sr, and PAA-Zn hydrogels

In a typical experiment, 0.25 g Na_2_SiO_3_ was dissolved in 10 mL of 3.125 wt% PAA (Mw = 250000) to adjust pH value of the solution. 2 mL of 3 mol/L CaCl_2_ (SrCl_2_ when preparing PAA-Sr; ZnCl_2_ when preparing PAA-Zn) was slowly dropped into the PAA solution using a pipette, under a vigorous stirring. A white sticky gel was formed. After stirring for another 15 min, the hydrogel was washed with distilled water for three times and stored in 50 mL of distilled water for two days.

#### 2.2.2 Mineralization of the hydrogels

0.5 g calcium hydroxide (Ca(OH)_2_) was dissolved in 100 mL water to prepare a supersaturated solution. The prepared PAA-Ca, PAA-Sr, and PAA-Zn hydrogels were immersed in the Ca(OH)_2_ solution for several days (0, 3, 7, and 14 days) and then rinsed with water before use.

#### 2.2.3 Preparation of decellularized bone matrix (DBM)

Firstly, native bone tissue was collected from a fresh porcine femur, and then, bone marrow, cartilage, and soft tissue were removed. The bone tissue was cleaned with a phosphate buffer solution (PBS) and chopped into smaller pieces. Then, the small bone tissue pieces were stirred in 0.5 M HCl (Yonghua Chemical Co., Ltd, Jiangsu) at room temperature for 72 h to decalcify. The decalcified bone matrix was placed in a 1:1 mixture of trichloromethane (Yonghua Chemical Co., Ltd, Jiangsu) and methanol (Aladdin, Shanghai) and agitated at 37 °C for 4 h. Then, the decalcified bone matrix was ground into powder by grinder. Finally, the decalcified bone matrix powder was decellularized by stirring in a solution of 0.05 % pepsin (Yuanye, Shanghai) and 0.02 % EDTA (Solarbio, Beijing) continuously for 24 h to obtain the final decellularized bone matrix.

#### 2.2.4 Preparation of DBM/hydrogel composites

Firstly, the prepared DBM was blended with hydrogel uniformly at a mass ratio of 1:10 to obtain DBM/hydrogel composites. Then the DBM/hydrogel composites were re-molded using PDMS molds. Finally, the DBM/hydrogel composites were immersed in Ca(OH)_2_ solution for further experiments.

### 2.3 Characterization

#### 2.3.1 Mechanical properties

PAA-Ca, PAA-Sr, and PAA-Zn hydrogels before mineralization were molded using a polydimethylsiloxane (PDMS, Dow Corning, American) cylinder with a diameter of 6 mm and a height of 13 mm. The hydrogels were immersed in ethanol for 1 min and demolded for subsequent mineralization. The surface of the hydrogels mineralized for 0, 3, 7, and 14 days was washed with deionized water and dried on filter paper. A universal testing machine (Shanghai Hengyi, China) was applied to measure the compressive strength of mineralized hydrogels at a vertical speed of 1 mm/min. Five specimens were tested for each group.

#### 2.3.2 Rheological properties

A Discovery HR2 Hybrid Rheometer (TA Instrument) was applied to measure the rheological properties of hydrogels. The hydrogels were placed on the device platform and aligned with the cone plate. A Peltier plate geometry (1000 μm gap, 20 mm plate diameter) was used to prepare hydrogels with a diameter of 20 mm. The temperature was kept at 37 °C. Dynamic frequency sweep from 0.1 to 100 s^−1^ was performed at a 1 % fixed strain. Then, the shear-thinning behavior of the hydrogels was examined by rheological measurements. The changes in shear stress and viscosity with the shear rate were measured at a shear rate from 0.1 to 100 s^−1^ at 37 °C. Finally, the influence of temperature on the rheological properties of hydrogels was investigated in the temperature range from 25 to 85 °C at a constant frequency of 1 s^−1^.

#### 2.3.3 Scanning electron microscopy (SEM)

Hydrogels before and after mineralization were cut into round slices and freeze-dried. The microstructure and elemental composition of the hydrogels were determined using a scanning electron microscope with an EDX detector (SEM, Hitachi Regulus 8100, Japan). The round slices were mounted on Al stubs using a double-sided carbon tape and sputtered with a thin Au coating for 60 s. The acceleration voltage was set to 15 kV.

#### 2.3.4 Atomic force microscopy (AFM)

Mineralized hydrogels were rinsed using deionized water and freeze-dried. Then, the freeze-dried hydrogels were cut into rectangular blocks (1 cm × 1 cm × 1 mm) for AFM analysis (AFM; Bruker Dimension ICON, Germany) to characterize the surface morphology and microstructures of the mineralized hydrogels. The contact mode of AFM was used to assess surface morphology.

#### 2.3.5 Transmission electron microscopy (TEM)

A JEOL JEM 2100F TEM was used to reveal the structure of mineralized hydrogels and elemental composition. Mineralized hydrogels were freeze-dried and grounded into powders in an agate mortar. Then, the powders were dispersed using ethanol, and a copper grid was dipped in the suspension. Selected area electron diffraction (SAED) and TEM images of hydrogels were obtained with a Philips CM200 microscope operating at 200 kV.

#### 2.3.6 Thermogravimetric analysis (TGA)

A thermal analyzer (SDT Q600 V20.9, USA) was used to perform thermogravimetric analysis under a nitrogen atmosphere. Hydrogels were freeze-dried and grounded into fine powders in an agate mortar. For each experiment, 5–6 mg of sample was added in an alumina crucible and placed on the thermobalance. The powders were heated from room temperature to 800 °C at a heating rate of 10 °C min^−1^.

#### 2.3.7 Fourier transform infrared spectroscopy (FTIR)

The mineralized hydrogel was freeze-dried and grounded into fine powder. Then, the powder and KBr were mixed at a ratio of 1:100 and grounded evenly using agate mortar. The mixed powder was pressed to KBr pellets using a pressure of 8 MPa. The KBr pellets was measured using FTIR (IS50, Thermo Scientifc) and the spectra was acquied from 400 to 4000 cm^−1^.

#### 2.3.8 X-ray photoelectron spectroscopy (XPS)

The aluminum foil (length: 1 cm, width: 1 cm) was wiped using absolute ethyl alcohol and stuck onto double-sided tape. Subsequently, the powder of mineralized hydrogel was spread on the tape. The powder was covered with another aluminum foil and then pressed using a pressure of 10 MPa for 10 s. Finally, the upper aluminum foil was removed and the sample was characterized using X-ray photoelectron spectroscopy (ESCALAB Xi+, Thermo Scientifc).

### 2.4 The biocompatibility and osteogenesis of mineralized hydrogel

The biocompatibility and osteogenesis of mineralized hydrogel were measured using live-dead staining and alkaline phosphatase (ALP) staining, respectively. The extracts were prepared using mineralized hydrogel and medium (complete medium or osteogenic induction medium) at a ratio of 0.2 g/mL. Firstly, the bone mesenchymal stem cells (BMSCs) were extracted from 6 to 8 weeks SD rats. The BMSCs were cultured with extracts for 3 days and then stained using the Live-dead kit. The fluorescent images were captured using inverted fluorescent microscope (Zeiss, Germany). The ALP staining was performed after the culture of BMSCs with extracts for 7 days. The images of ALP staining were obtained using optical microscope.

### 2.5 RNA extraction and real-time quantitative PCR (RT-qPCR)

Cells were cultured with mineralized hydrogels for 7 days. Then, the total RNA extracted by TRIzol (Beyotime, Shanghai, China) was measured by a NanoDrop 2000 spectrophotometer and used to reverse-transcribe into cDNA with RT Master Mix (RR036Q, Takara Bio). The obtained cDNA was diluted to 10 ng/μL with nuclease-free water (Beyotime, Shanghai, China). The qPCR reaction components included 1 μL forward primer, 1 μL reverse primer, 1.5 μL nuclease-free water, 2.5 μL cDNA, and 5 μL Master Mix. PCR cycle steps were as follows: Stage 1: pre-denaturation at 95 °C for 30 s; Stage 2: denaturation, repeated 40 times at 95 °C for 5 s; Stage 3: annealing and extension at 60 °C for 30 s. The primers were synthesized by Sangon Biotech (Shanghai, China), and the primer sequences in this study are presented in Table S1.

### 2.6 Western blot analysis

BMSCs were treated with α-MEM (Control group), DBM, M-PAA-Ca, and M-PAA-Sr hydrogels for 7 days. The total proteins from BMSCs were extracted with a RIPA Lysis Buffer (Solarbio, Beijing, China). The concentration of protein in each group was determined using a BCA protein assay kit (Yeasen, Shanghai, China). An SDS-PAGE loading buffer (EpiZyme, Shanghai, China) was used to mix with 15 μg protein lysate of each sample, denatured at 100 °C for 5 min, separated on 10 % SDS–PAGE gels, and then electroblotted onto nitrocellulose (NC) membranes (Beyotime, Shanghai, China). 5 % non-fat milk was used to block the samples for 2 h. The primary antibodies (diluted 1:1000) were used to incubate these membranes at 4 °C overnight and then treated with HRP-conjugated secondary antibodies (Beyotime, Shanghai, China) at room temperature for 1 h. The protein bands were visualized using a chemiluminescence imaging system (SHST, Hangzhou, China). Primary antibodies were ColⅠ (Abcam, Cambridge, UK) and Runx2 (Cell Signaling Technology, MA, USA).

### 2.7 RNA-seq analysis

BMSCs were treated with α-MEM (Control group), DBM, M-PAA-Ca, and M-PAA-Sr hydrogels for 7 days. Three parallel samples were set in each group. Trizol reagent (Vazyme, Nanjing, China) was used to obtain the total RNA from different groups. Transcriptome sequencing and analysis were conducted by LC Bio Technology CO, Ltd (Hangzhou, China). HISAT2 software was used to map reads to the reference genome of the Rattus genome. StringTie software was used to determine the expression level of mRNAs by calculating FPKM. The expressed genes with fold change >2 and p-value <0.05 were considered differentially expressed genes (DEGs). Gene ontology (GO) analysis and Kyoto encyclopedia of genes and genomes (KEGG) analysis were performed with DAVID software (https://david.ncifcrf.gov/). Gene set enrichment analysis (GSEA) was obtained from OmicStudio cloud platform (https://www.omicstudio.cn/home).

### 2.8 Rat calvarial critical-size defect model and scaffold implantation

All procedures followed the NIH Guide for the Care and Use of Laboratory Animals and were approved by the Ethics Committee of Soochow University (SUDA20220913A02). The rats’ cranial defect model was established to evaluate the effect of mineralized hydrogels on promoting bone regeneration (Ctrl group, ECM group, M-PAA-Ca group, M-PAA-Sr group). The SD male rats (number: 36, 8–10 weeks old, 300–350 g) were anesthetized under intraperitoneal injection of 1.5 % pentobarbital sodium (30 mg per kg rat body weight). The cranial defects (5 mm in diameter) were constructed using a micro bone drill, and the mineralized hydrogels were placed into the constructed bone defect areas. After that, the skin was tightly sutured. The rats were sacrificed at 2, 4, and 8 weeks post operation, and the skulls were harvested and fixed with 10 % formalin solution. The number of ECM group, M-PAA-Ca group, M-PAA-Sr group samples were 25, respectively.

### 2.9 Micro-computed tomography analysis (Micro-CT analysis)

The cranials harvested from the operated rats were fixed using a 10 % formalin solution for 48 h and then subjected to radiographic analysis using a micro-CT scanner (SkyScan 1176, SkyScan, Belgium). The micro-CT scanner was set at 100 kV, 100 mA, and 1 mm aluminum filter. The scanned images were reconstructed using Skyscan NRecon software. Following reconstruction, the images were set up at the region of interest to operate Data viewer and SkyScan CTAn for visualizing the newly formed bone and the surplus of implant material, and 3D images were processed using Mimics software.

### 2.10 Histological evaluation

The harvested cranials were decalcified in 10 % ethylenediaminetetraacetic acid (EDTA) for 4 weeks. The EDTA solution was changed every day. Then, the samples were dehydrated with a gradient concentration (75, 80, 90, 95, and 100 %) of ethanol and embedded in paraffin. The embedded cranials were sectioned into tissue slides with a thickness of 7 μm using a micro-tome (LEICA, German), and then hematoxylin and eosin (H&E) and Masson's trichrome staining procedures were used for histological analysis. First, tissue samples were deparaffinized to remove embedding paraffin. Subsequently, the deparaffinized samples were rehydrated through a graded ethanol series (100 % anhydrous ethanol→95 % ethanol→80 % ethanol). The rehydrated samples were stained using H&E (Hematoxylin and Eosin), Masson's Trichrome (ponceau S and aniline blue). Following staining, samples are dehydrated through ethanol gradient (80 %→95 %→100 % ethanol). Finally, tissues are cleared in xylene and permanently mounted with resinous medium.

### 2.11 Immunofluorescent staining

2 weeks post operation, the harvested cranials were fixed in 10 % formalin for 24 h, washed with PBS, and decalcified in 10 % EDTA for 1 month. Next, the samples were dehydrated with a gradient concentration (75, 80, 90, 95, and 100 %) of ethanol and embedded in paraffin. The embedded samples were sectioned into tissue slides with a thickness of 6 μm using a micro-tome (LEICA, German). Samples were stained with CD206 (1:300, Cat. No. sc-58986, Santa Cruz, CA, USA), CD80 (1:200, Cat. No. A16039, ABclonal, MA, USA), and CD31 (1:100, Cat. No. ab222783, Abcam, Cambridge, UK) to evaluate vascularization and macrophage polarization, respectively. A high-resolution fluorescence microscope (Zeiss Axiovert 200, USA) was applied to assess the expression of CD31, CD80, and CD206. Image J software was used for quantitative analysis.

### 2.12 Statistical analysis

Quantitative data were presented as the mean value ± standard deviation. The differences between two groups were compared using the Student's t-test, and a one-way analysis of variance (ANOVA) followed by Tukey's test was performed to make multiple comparisons (OriginLab Corporation, MA, USA). A value of p < 0.05 denoted a statistically significant difference.

## 3 Results and discussion

### 3.1 Preparation and characterization of the mineralized hydrogels

**Fig. 1:**
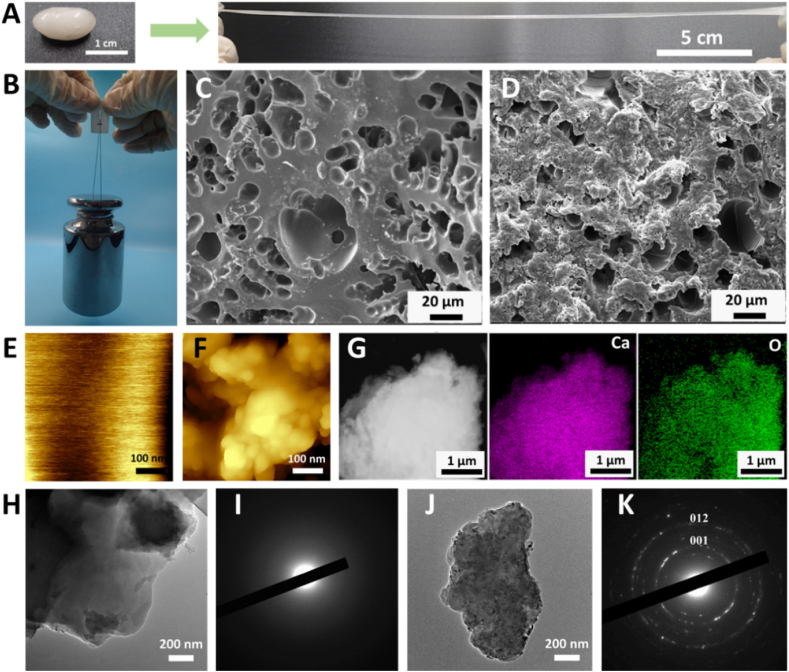
Structural and compositional characterization. (A) The PAA-Ca hydrogel is stretchable before mineralization. (B) The mineralized hydrogel can hold a load of 1 kg. (C–D) SEM images of the freeze-dried hydrogels before (C) and after mineralization (D). (E–F) AFM images of the freeze-dried hydrogels before (E) and after mineralization (F). (G) EDX mapping of the mineralized hydrogel. (H–I) The TEM image (H) and the SAED pattern (I) of the hydrogel before mineralization. (J–K) The TEM image (J) and the SAED pattern (K) of the hydrogel after mineralization.

In previous studies, the hydrogels were obtained by physically crosslinking amorphous calcium carbonate (ACC) with polyacrylic acid chains [24], or through the crosslinking between deprotonated PAA chains and Ca^2+^ [39]. In this study, we used an alternative synthetic route, through which larger amount of hydrogels can be prepared. In a typical experiment, a solution consisting of 10 mL of 3.125 % PAA (Mw = 250000) and 0.25 g Na_2_SiO_3_**·**5H_2_O was first prepared, then 2 mL of 3 mol/L CaCl_2_ was dropped into the solution under vigorous stirring using a pipette. With the addition of CaCl_2_, the solution turned turbid, and later a gel-like substance formed. After washing and storage in water for 2 days, a semi-transparent gel was prepared. The obtained hydrogel was shapeable and stretchable, and the length of the stretched hydrogel was up to 15 times higher than its original length (Fig. 1A and Fig. S1A). In addition, the hydrogel exhibited great self-healing properties (Fig. S1B). The rheological properties of the hydrogels were investigated through dynamic frequency sweep and thixotropic experiments. The dynamic storage modulus (G′) and the loss modulus (G″) varied with the angular frequency, indicating physical crosslinking of the hydrogels. G′ and G″ were similar, implying the semi-liquid state of the hydrogels (Fig. S2A). The hydrogels exhibited shear-thinning behavior, with viscosity decreasing with the shear rate (Fig. S2B). Moreover, G′ and G″ were greatly affected by temperature, i.e., both G′ and G″ increased with temperature, and G′ was higher than G″, indicating a harder and more solid-like hydrogel (Fig. S2C).

The PAA-Ca hydrogel before mineralization was mechanically weak. After immersing in the supersaturated Ca(OH)_2_ solution for 14 days, the hydrogel became strong, with the capacity to withstand a 1 kg load (Fig. 1B). The sample was freeze-dried and the microstructure inside the hydrogel was analyzed. The surface of the hydrogel was relatively smooth, with a porous structure inside (Fig. 1C and E). After mineralization, the surface became rough and the mineral phase could be observed (Fig. 1D and F). An energy dispersive X-ray (EDX) mapping showed, the mineral phase mainly consisted of calcium and oxygen (Fig. 1G). The X-ray diffraction (XRD) patterns showed that the hydrogels before and after mineralization were mainly amorphous (Fig. S3). However, although the transmission electron microscopy (TEM) images and selected area electron diffraction (SAED) patterns confirmed that the hydrogel before mineralization was amorphous, calcium hydroxide with low crystallinity was identified in the hydrogels after mineralization (Fig. 1H–K). The FTIR analysis revealed characteristic peaks of COO^−^ groups at approximately 1420 cm^−1^ and 1620 cm^−1^ (Fig. S4). XPS further confirmed the presence of peaks corresponding to C 1s, Ca 2p, and O 1s over a wide binding energy range. (Fig. S5).

**Fig. 2:**
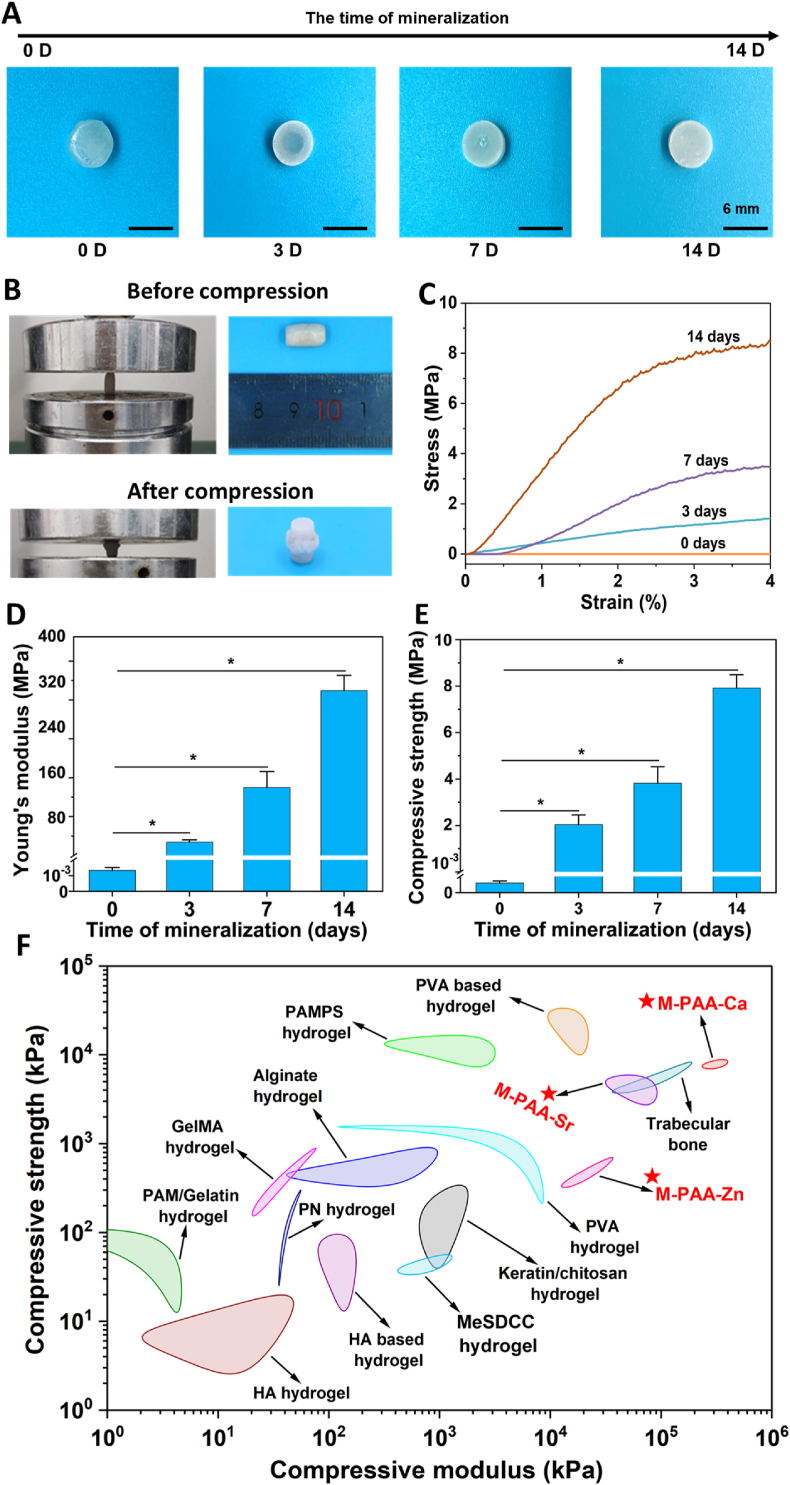
(A) Digital photographs of PAA-Ca hydrogels before and after 3, 7, and 14 days of mineralization. (B) Digital images of mineralized hydrogels (14 days) before and after compression. (C) Compressive stress-strain curves of the hydrogels. (D) Elastic modulus of the hydrogels (n = 5). (E) Compressive strength of the hydrogels (n = 5). (F) A schematic showing the comparison of compressive strength and elastic modulus of the mineralized hydrogels and those of natural bone and other hydrogels (Our work is compared to other reported tough materials, including GelMA hydrogels [40], PVA hydrogels [41], PVA based hydrogels [42,43], HA based hydrogels [44,45], Keratin/chitosan hydrogels [46], MeSDCC hydrogels [47], PAMPS hydrogels [48], Alginate hydrogels [49], PN hydrogels [50], PAM and gelatin hydrogels [51], and Cellulose hydrogels [52]).

The mineralization process and its effect on the mechanical strength of the hydrogel were further investigated. The mineralization occurred from outside to inside, and the hydrogel turned from transparent to opaque after mineralizing for 14 days (Fig. 2A). The hydrogel after mineralization exhibited a plastic failure mode during compressive testing, and the mineralization dramatically improved the mechanical performance of the hydrogels (Fig. 2B and C). The compressive strength and modulus of the hydrogels before mineralization were 0.3 ± 0.1 kPa and 1.3 ± 0.2 kPa, and they reached 7.9 ± 0.6 MPa and 339.3 ± 31.4 MPa, respectively, after mineralization for 14 days (Fig. 2D and E). The values were higher than for most of reported hydrogels, and more importantly, they were very similar to the mechanical properties of trabecular bone (Fig. 2F). The materials applied for the replacement of trabecular bone should have similar mechanical properties, especially the elastic modulus. The results of compressive tests demonstrated that the applied mineralization procedure dramatically enhanced the mechanical properties of the hydrogels, enabling their application for load-bearing bone repair. The TGA curves before and after mineralization were similar, however, the mineralized sample showed a higher content of remaining material, indicating a higher mineral content (Fig. S6). It is worth noting that the strengthening effect is not only applicable to PAA-Ca but also to hydrogels prepared with Sr and Zn. By replacing CaCl_2_ with ZnCl_2_ and SrCl_2_, PAA-Sr and PAA-Zn were prepared. Then, the hydrogels were immersed in super saturated Ca(OH)_2_ solution for strengthening. Similar to PAA-Ca, the compressive strength of PAA-Sr and PAA-Zn increased to 4.3 ± 1.0 MPa and 1.6 ± 0.6 MPa, respectively (Fig. S7A), and their elastic moduli reached up to 61.8 ± 18.7 MPa and 23.3 ± 7.6 MPa after mineralization (Fig. S7B). The EDX results revealed the presence of calcium and strontium in M-PAA-Sr, and the presence of calcium and zinc in M-PAA-Zn (Fig. S8).

### 3.2 In vivo bone formation

**Fig. 3:**
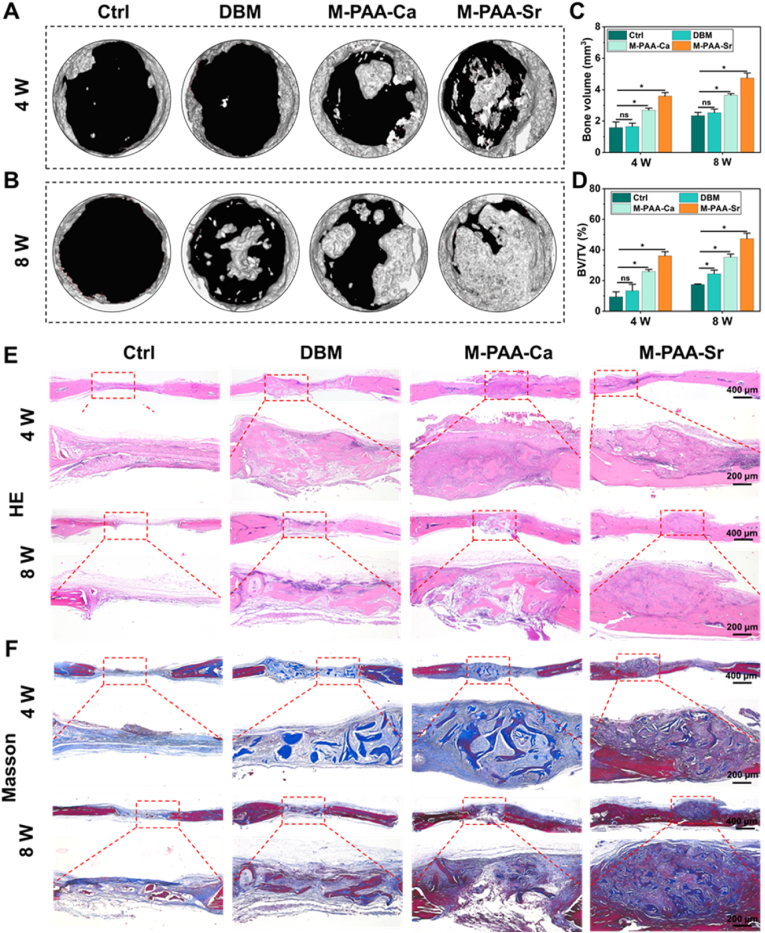
(A–B) 3D reconstructed micro-CT images of the newly formed bone in rat skull defects at weeks 4 and 8 postoperatively. (C–D) Quantitative analysis of the microstructural parameters of bone volume (BV) and bone volume/tissue volume (BV/TV) ratios at the defect sites. (E–F) Low and high magnification images of H&E staining and Masson's trichrome staining of new bone formation at the defect site 4 and 8 weeks after implantation. The error bars represent the standard deviation obtained from measurements of n = 16 independent samples from 16 rats, each with four independent repeats. Statistical analysis was performed using ordinary one-way ANOVA tests with OriginLab. ∗, *p* < 0.05.

Encouraged by the superior mechanical properties, the in vivo osteogenic properties of the mineralized hydrogels were evaluated using the cranial defect model of rats. Demineralized bone matrix (DBM) is osteoconductive and osteoinductive, and it was extensively applied in the clinical practice for bone regeneration [37]. However, the use of DBM alone in load-bearing conditions is contraindicated because of its mechanical weakness. In this study, we blended DBM (Fig. S9) with the hydrogels and mineralized for 14 days to fabricate bone grafting materials with good bioactivity and structural rigidity. Calcium is one of the most abundant elements in the human body and strontium promotes new bone formation by affecting both osteoblasts and osteoclasts. Live/Dead staining demonstrated near-complete cell viability in all modified hydrogel groups (M-PAA-Ca, M-PAA-Sr), with minimal dead cell detection. Cytoskeleton staining showed that the cells are well-spread. Furthermore, ALP staining showed significantly higher enzymatic activity in M-PAA-Ca and M-PAA-Sr. These results of staining demonstrated that both M-PAA-Ca and M-PAA-Sr exhibited excellent biocompatibility and promoted osteogenesis (Figs. S10, S11, S12). Therefore, PAA-Ca and PAA-Sr hydrogels are chosen for the exploration. Micro-CT was taken at weeks 4 and 8 after implantation of the DBM/hydrogel composites. As shown in Fig. 3A and B, the control and DBM groups showed delayed fracture healing, while M-PAA-Ca and M-PAA-Sr groups exhibited superior bone formation by the end of weeks 4 and 8. Quantitative analysis showed that the bone volume (BV) and bone volume/tissue volume (BV/TV) were significantly higher in M-PAA-Ca and M-PAA-Sr groups than in control (1.58 ± 0.28; 9.41 ± 2.59) and DBM groups at 4 weeks (Fig. 3C). After 8 weeks, the highest BV and BV/TV of new bone formation was found in the M-PAA-Sr group (Fig. 3D). These results demonstrated the superior regeneration of new bone tissue by M-PAA-Sr compared to other groups. Representative hematoxylin and eosin (H&E) staining and Masson's trichrome staining sections confirmed that the new bone formation was accelerated in M-PAA-Ca and M-PAA-Sr groups (Fig. 3E and F). The DBM was visible at week 4, and it was gradually replaced by mature bone tissue 8 weeks after implantation. Moreover, a higher amount of mature bone was observed at the defect site induced by M-PAA-Sr than M-PAA-Ca at weeks 4 and 8 (Fig. 3F), confirming the superior osteogenic property of strontium-containing composites. Calcium is the main component of human bone and it can stimulate the formation of osteoblasts during the bone regeneration. Strontium, as a homologous element to calcium and an important trace element in bone tissue, was demonstrated to activate the Wnt signaling pathway, regulating proliferation, differentiation, and mineralization of bone marrow mesenchymal stem cells [53]. Additionally, strontium efficiently inhibited the formation and differentiation of osteoclasts. In this study, we have shown that the PAA-Sr hydrogel/DBM composites can facilitate the bone formation process.

**Fig. 4:**
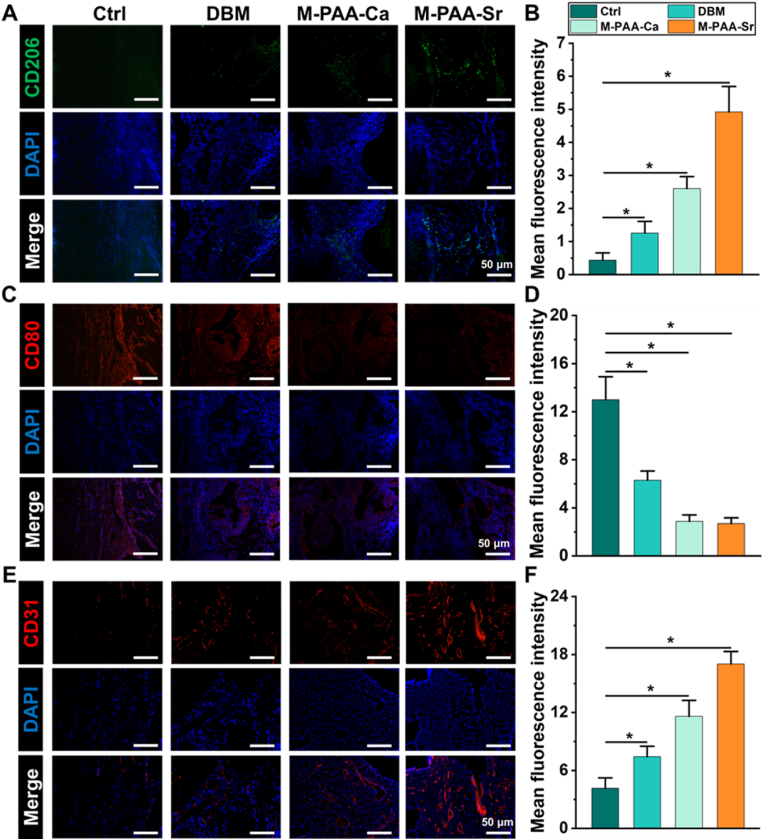
Immunofluorescent staining of cranials at 2 weeks. (A) Immunofluorescence staining images of cross-sections of grafts stained with CD206 (M2 marker). (B) Quantitative analysis of the expression level of CD206. (C) Immunofluorescence staining images of cross-sections of grafts stained with CD80 (M1 marker). (D) Quantitative analysis of the expression level of CD80. (E) Immunofluorescent staining images for CD31 of neovascularization at the defects sites. (F) Quantitative analysis of the expression level of CD31. The error bars represent the standard deviation obtained from measurements of n = 12 independent samples from 12 rats, each with three independent repeats. Statistical analysis was performed using ordinary one-way ANOVA tests with OriginLab. ∗, *p* < 0.05.

The immune microenvironment at the bone defect site plays an essential role in early angiogenesis and bone regeneration. In previous studies, Sr^2+^ was reported to regulate macrophages polarization from M1-type to the M2-type and build a suitable local microenvironment to stimulate angiogenesis, which could further enhance osteogenesis [54,55]. To clarify better immune-regulation effects of M-PAA-Sr than M-PAA-Ca, the expression of macrophage markers (M1:CD80, M2:CD206) and blood vessel marker (CD31) were detected by immunofluorescent staining. As shown in Fig. 4A–D, in the M-PAA-Ca and M-PAA-Sr groups, the amount of CD206 expressing macrophages was significantly lower (Fig. 4A and B), and the number of CD80 expressing macrophages was significantly higher (Fig. 4C and D) than in DBM and control groups. In comparison to the M-PAA-Ca group, the M-PAA-Sr group better promoted the polarization from M1 to M2 macrophages. Moreover, the M-PAA-Sr group showed the highest expression of CD31 (Fig. 4E and F), which is an essential marker for vascular endothelial differentiation [56,57]. Our results demonstrated that the mineralized hydrogels, especially the M-PAA-Sr hydrogel, can polarize macrophages toward the M2 phenotype and promote the angiogenesis at an early stage of the bone healing process.

### 3.3 Potential signaling pathway for the therapeutic effects of the composites

**Fig. 5:**
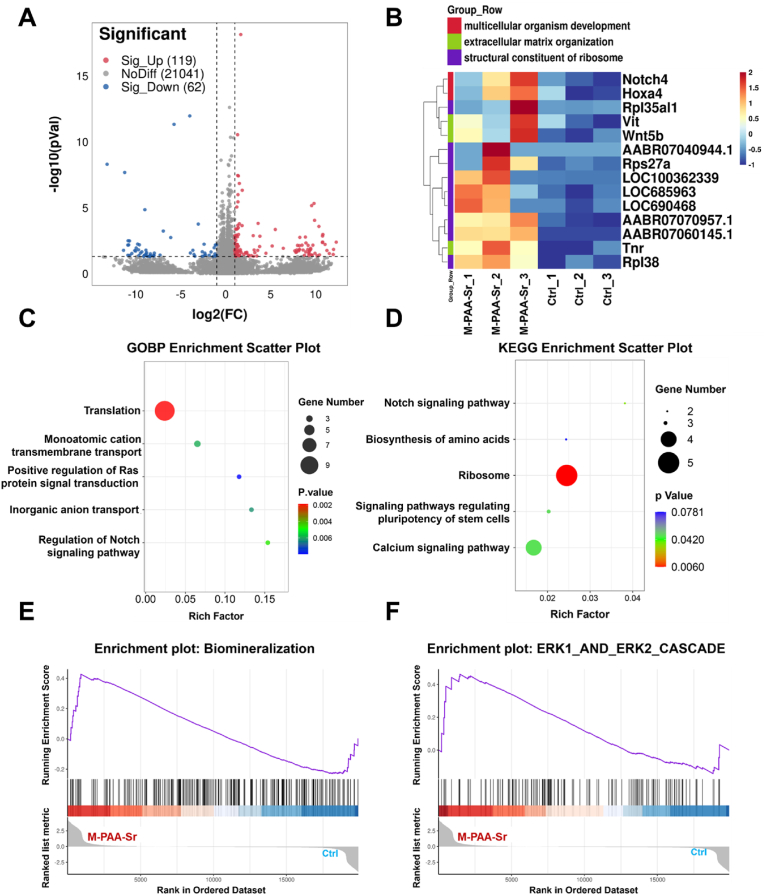
Transcriptomic analysis. (A) Volcano plot of differentially expressed genes (DEGs). Red dots indicate upregulated genes; Blue dots indicate downregulated genes; Grey dots indicate unchanged genes. (B) The heatmap shows a series of DEGs related to multicellular organism development, extracellular matrix organization, and structural constituent of ribosome in BMSCs (n = 3). (C) GO analysis shows the main biological processes involved in DEGs. (D) KEGG enrichment bubble plot shows the main pathways involved in DEGs. (E–F) GSEA shows that the “Biomineralization” and “ERK1_and ERK2_CASCADE” were upregulated in the M-PAA-Sr group. (For interpretation of the references to colour in this figure legend, the reader is referred to the Web version of this article.)

To further determine the underlying mechanism of enhanced bone regeneration for the M-PAA-Sr composite, RNA-seq was conducted to explore differentially expressed genes (DEGs) between BMSCs in M-PAA-Sr and Ctrl groups after culturing for one week. Compared with the Ctrl group, 119 genes were significantly upregulated, and 62 genes were significantly downregulated in BMSCs cultured on M-PAA-Sr, as shown in a volcano plot (|log2FC| ≥ 1 & p < 0.05) (Fig. 5A). The heatmap revealed that the featured biomarkers related to multicellular organism development, extracellular matrix organization, and structural constituent of ribosome were basically upregulated in thee PAA-Sr group (Fig. 5B). Gene ontology (GO) enrichment analysis was carried out to determine the functional changes underlying these genomic data, and a list of GO terms was generated accordingly. GO analysis showed that these DEGs were mainly associated with translation, inorganic anion/cation transport, Ras signaling pathway, and Notch signaling pathway (Fig. 5C). These biological processes participate in secretion of extracellular matrix, homeostasis of stem cells, and osteogenic differentiation. To identify potential signaling pathways involved in these processes, Kyoto Encyclopedia of Genes and Genomes (KEGG) pathway enrichment analysis was performed. The results showed that Ribosome, Notch signaling pathway, calcium signaling pathway, and signaling pathways related to pluripotency of stem cells were upregulated in the M-PAA-Sr group compared to the Ctrl group (Fig. 5D).

Ribosomes are complex ribozymes that translate messenger RNA into proteins and participate in matrix synthesis [58]. The Notch signaling pathway controls stem cell behavior and is responsible for regulation of stemness maintenance, generation efficacy, and specific lineage differentiation of stem cells [59]. Sr^2+^ was reported to balance stemness maintenance and differentiation through asymmetric cell division, and the increase in stem cells population is favorable for enhancing osteogenic differentiation [60]. The activation of cation channels is believed to activate Ras, and subsequently stimulates a series of pathways, such as the MEK1/2-ERK1/2 cascade, which plays an essential role in osteogenic differentiation [61,62]. In accordance with these studies, Gene Set Enrichment Analysis (GSEA) demonstrated that the “biomineralization” and “ERK1 and ERK2 cascade” were all markedly upregulated in the M-PAA-Sr group (Fig. 5E and F). Biomineralization is a biological process in which hydroxyapatite deposits onto collagen fibrils especially collagen type I (Col1a1) of the extracellular matrix [63,64]. In order to illustrate the effect of ribosome and translation in biomineralization, the expression of Col1a1 and its upstream transcription factor runt-related protein 2 (Runx2) were measured by qPCR and western blot. As shown in Fig. S13A, there was no obvious difference in the mRNA level of Runx2 and Col1a1 in each group. Interestingly, the protein level of Runx2 was slightly increased, while the protein level of Col1a1 was sharply raised in the M-PAA-Sr group, confirming that enhanced translation boosted the expression of matrix related to biomineralization (Fig. S13B). These results were also consistent with the in vivo experiment that numerous matrices were found in the M-PAA-Sr group, which might provide template for biomineralization. It is therefore assumed that M-PAA-Sr promoted the osteogenesis of BMSCs via at least three possible ways: increasing stem cells population and pluripotency through the Notch signaling pathway, triggering osteogenic differentiation through cation channels and ERK1/2 activation, and enhancing extracellular biosynthesis and biomineralization through translation.

## 4 Conclusions

In summary, we proposed a cost-effective way to fabricate mechanically robust and highly osteogenic bone-grafting materials via in situ mineralization of hydrogels. The hydrogels, which consist of polyacrylic acid chains cross-linked by bivalent cations, are stretchable and have the ability to self-heal, but they have very low mechanical strength (compressive strength 0.3 ± 0.1 kPa, elastic modulus 1.3 ± 0.2 kPa). Controlled mineralization of the synthesized hydrogels in a Ca(OH)_2_ solution has led to in situ formation of nano calcium hydroxide crystals, and materials with exceptional mechanical properties were fabricated, showing the compressive strength and modulus of 7.9 ± 0.6 MPa and 339.3 ± 31.4 MPa, respectively, surpassing those of trabecular bone. Furthermore, hydrogel/DBM composites were mineralized and after implanted of into the rat calvarial critical-size defects. The composites enhanced fracture healing at weeks 4 and 8. The enhanced bone healing can be attributed to the polarization of macrophages towards the M2 phenotype and the abundant formation of new blood vessels induced by the composites. Transcriptome sequencing revealed that the composites promoted osteogenesis through enhancing extracellular biosynthesis and biomineralization during the translation process. Through these characteristics, we demonstrated the applicability of mineralized hydrogel to enhance hard tissue regeneration, especially under load-bearing conditions.

## CRediT authorship contribution statement

**Song Chen:** Writing – original draft, Supervision, Investigation, Funding acquisition. **Dachuan Liu:** Visualization, Validation, Methodology, Data curation. **Qianping Guo:** Writing – original draft, Formal analysis, Data curation. **Li Dong:** Software, Resources. **Huan Wang:** Software, Resources, Formal analysis. **Jiaxu Shi:** Validation, Formal analysis, Data curation. **Weicheng Chen:** Resources, Methodology. **Caihong Zhu:** Resources, Methodology, Investigation. **Weishan Wang:** Formal analysis, Data curation. **Wei Xia:** Resources, Methodology, Conceptualization. **Miodrag J. Lukic:** Writing – review & editing, Visualization, Conceptualization. **Helmut Cölfen:** Methodology, Investigation, Conceptualization. **Bin Li:** Investigation, Funding acquisition, Conceptualization.

## Funding

This work was supported by 10.13039/501100001809National Natural Science Foundation of China (32271421, 81925027, 82002275), 10.13039/501100017526International Cooperation Project of Ningbo City (No. 2023H013), and the 10.13039/501100012246Priority Academic Program Development of Jiangsu Higher Education Institutions. MJL thanks the Ministry of Science, Technological Development and Innovation of the Republic of Serbia (Contract No: 451-03-66/2024-03/200017). This study is dedicated to the memory of Prof. Helmut Cölfen.

## Declaration of competing interest

The authors declare that they have no known competing financial interests or personal relationships that could have appeared to influence the work reported in this paper.

## Data Availability

Data will be made available on request.
