# Supplementary material for: Facile construction of mechanically robust and highly osteogenic materials for bone regeneration
Source: Mater Today Bio. 2025 May 3;32:101809. doi: 10.1016/j.mtbio.2025.101809 (PMC12190205; doi:10.1016/j.mtbio.2025.101809)
Supplement: Multimedia component 1 [file mmc1.docx]

**Supporting Information**

Facile construction of mechanically robust and highly osteogenic materials for bone regeneration

*Song Chen*^1#^*, *Dachuan Liu*^1^^#^*, Qianping Guo*^1#^, *Li Dong*^1^, *Huan Wang*^1^, *Jiaxu Shi*^1^, *Weicheng Chen*^1^, *Caihong Zhu*^1^, *Weishan Wang*^3^, *Wei Xia*^4^, *Miodrag J. Lukic*^5^, *Helmut Cölfen*^6^*, *Bin Li*^1,2^*

^1^ Medical 3D Printing Center, Orthopedic Institute, Department of Orthopedic Surgery, The First Affiliated Hospital, MOE Key Laboratory of Geriatric Diseases and Immunology, School of Basic Medical Sciences, Suzhou Medical College, Soochow University, Suzhou, Jiangsu 215000, P.R. China

^2^ Collaborative Innovation Center of Hematology, Soochow University, Suzhou, Jiangsu 215000, P.R. China

^3^ Department of Orthopaedic Surgery, The First Affiliated Hospital, Shihezi University School of Medicine, Shihezi, Xinjiang, P.R. China

^4^ Applied Materials Science, Department of Engineering Science, Uppsala University, Uppsala, Sweden

^5^ Laboratory of Physics, “Vinca” Institute of Nuclear Sciences, National Institute of the Republic of Serbia, University of Belgrade, Mike Petrovica Alasa 12-14, 11351, Vinca, Belgrade, Serbia

^6^ Physical Chemistry, Department of Chemistry, University of Konstanz, Universitätsstraße 10, 78457 Konstanz, Germany

^#^ The authors contribute equally to the paper

* Corresponding authors: Bin Li ([binli@suda.edu.cn](mailto:binli@suda.edu.cn)); Helmut Cölfen ([helmut.coelfen@uni-konstanz.de](mailto:helmut.coelfen@uni-konstanz.de)); Song Chen ([chensong@suda.edu.cn](mailto:chensong@suda.edu.cn)).


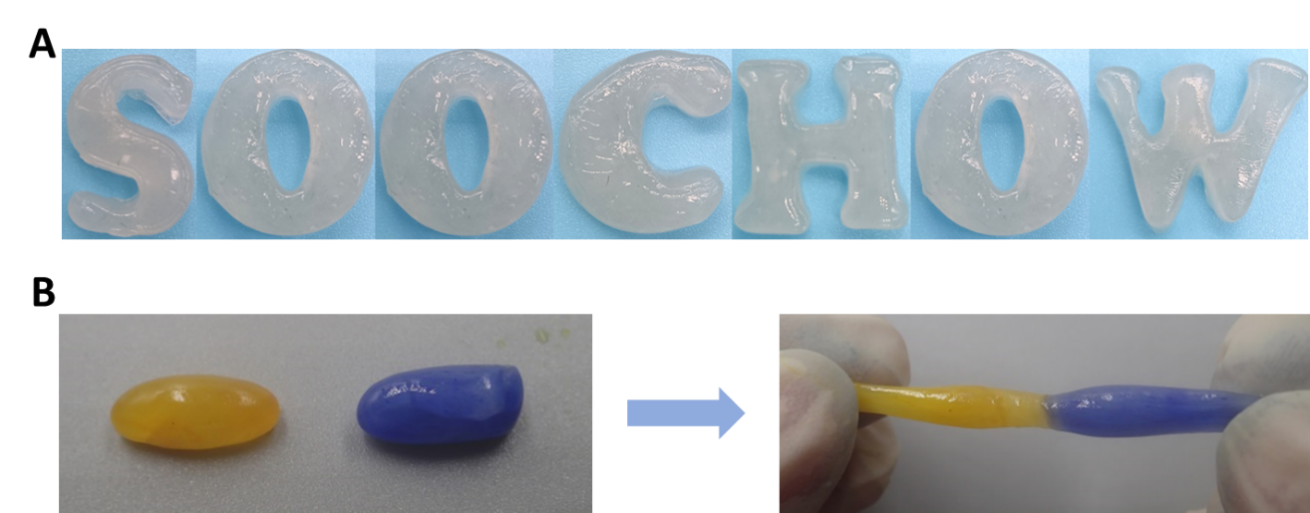


**Fig.S1.** The prepared PAA-Ca hydrogel can be molded into various shapes (A) and it is self-healing (B).


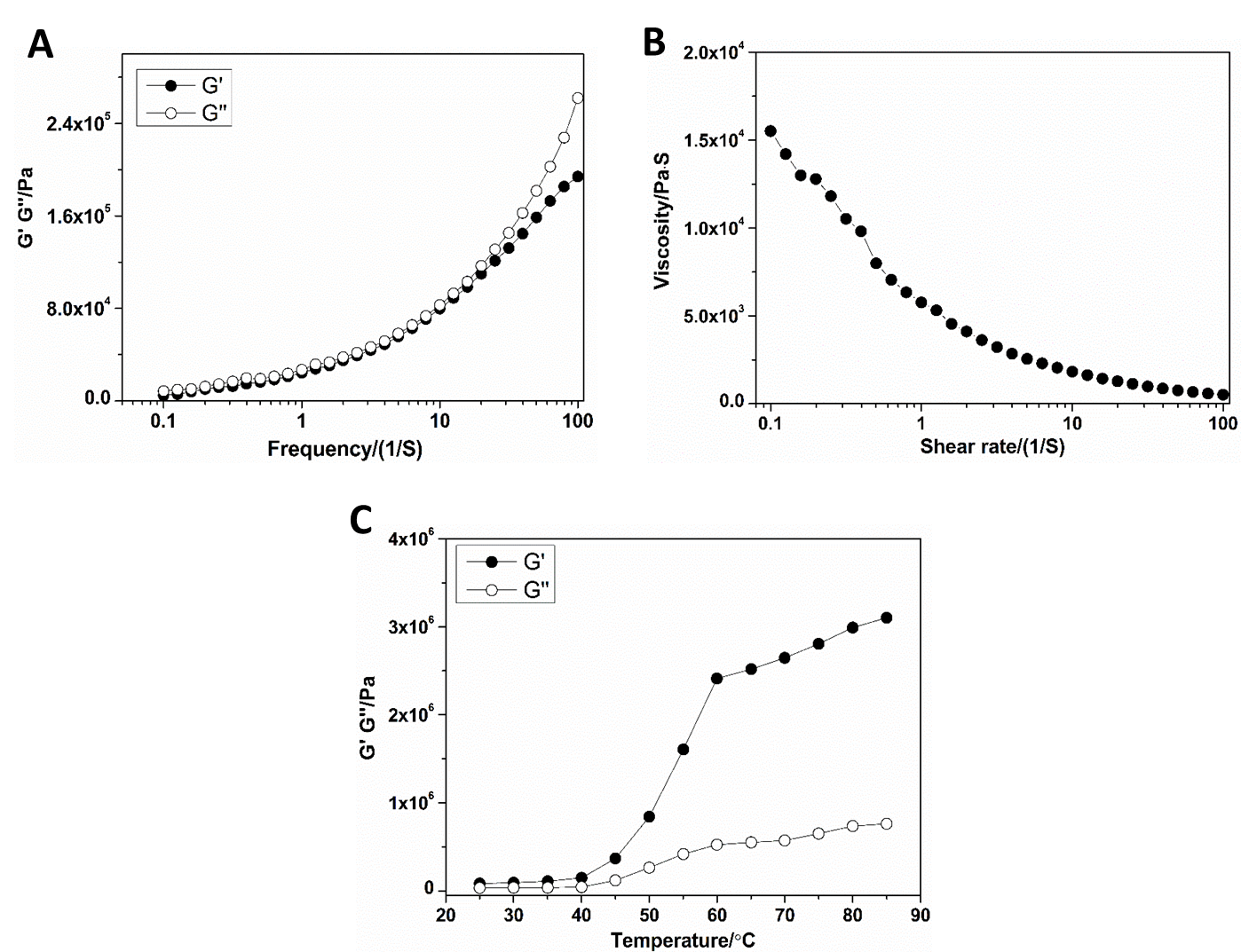


**Fig.S2.** (A) Frequency dependencies of the storage (G’) and loss (G’’) moduli of the PAA-Ca hydrogels before mineralization. (B) Change in viscosity with shear rate. (C) Change in storage (G’) and loss (G’’) moduli with temperature.





**Fig.S3.** X-ray diffraction pattern of the PAA-Ca hydrogel after mineralization.


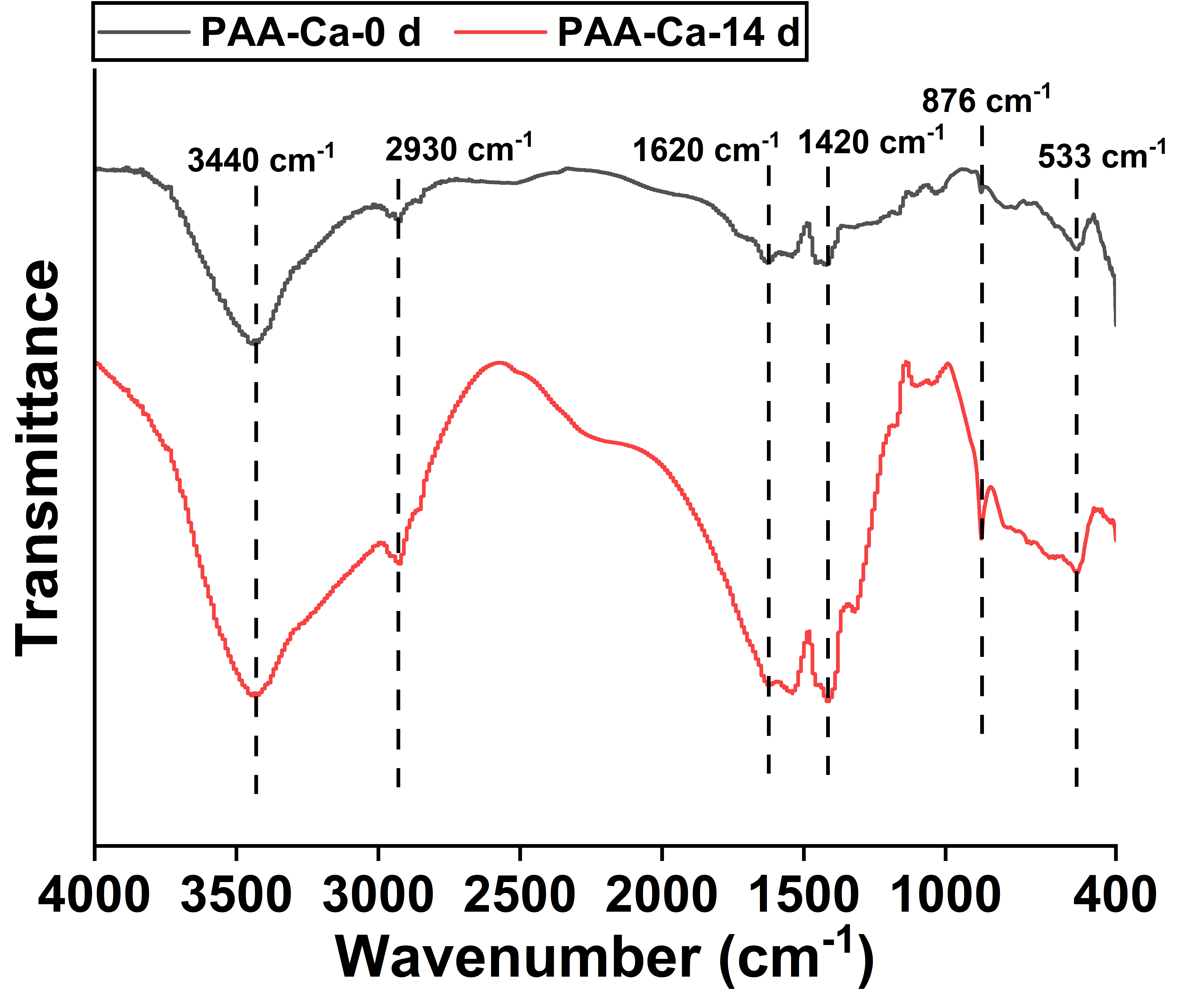


**Fig.S4.** Fourier transform infrared spectroscopy (FTIR) of the hydrogel after mineralization.


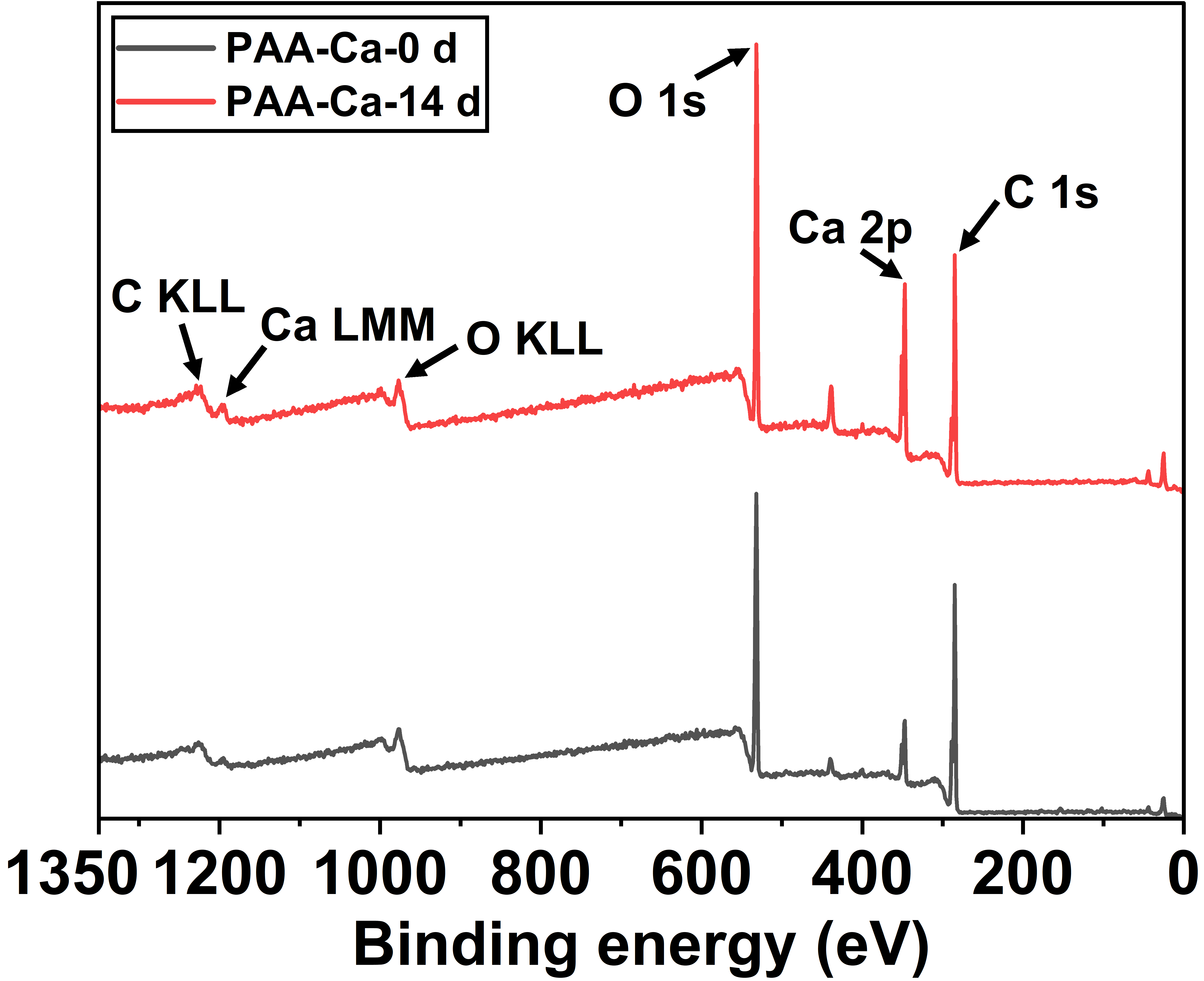


**Fig.S5.** X-ray photoelectron spectroscopy (XPS) of the hydrogel after mineralization.


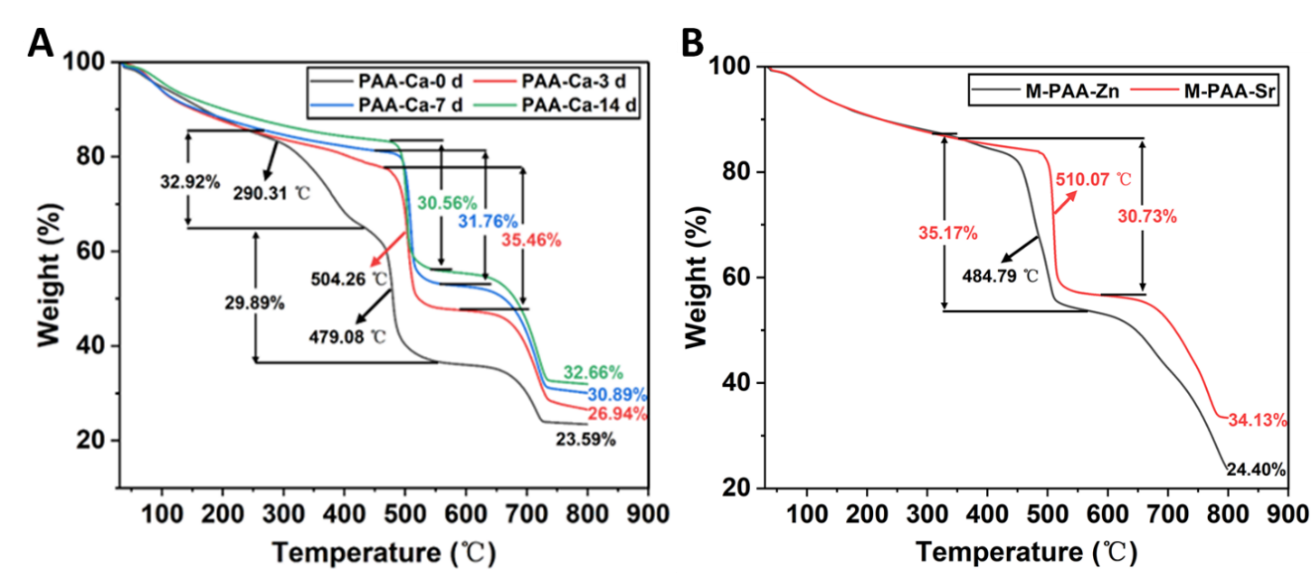


**Fig.S6.** TGA curve of the hydrogels. (A) PAA-Ca hydrogels before and after mineralization. (B) PAA-Zn and PAA-Sr hydrogels after mineralization for 14 days.


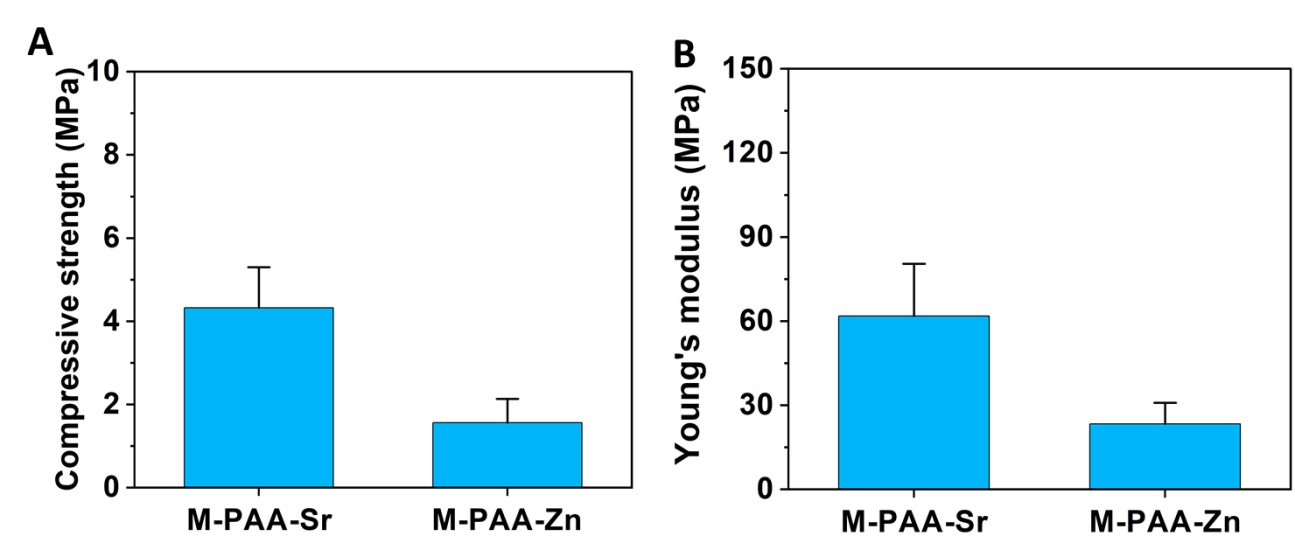


**Fig.S7.** Compressive strength and Young’s moduli of M-PAA-Sr (A) and M-PAA-Zn (B) (n=5).


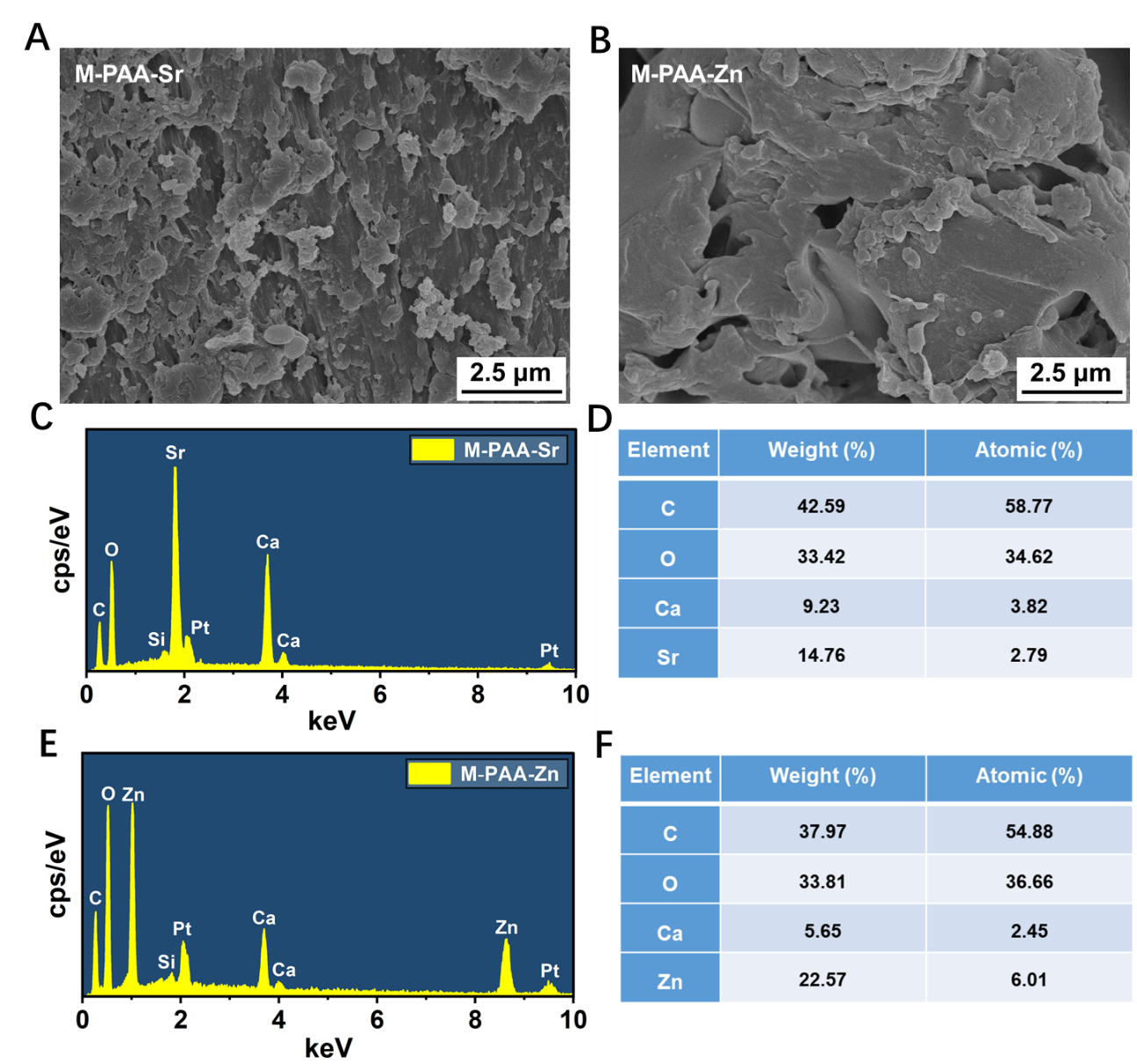


**Fig.S8.** Surface morphology of M-PAA-Sr (A) and M-PAA-Zn (B) after mineralization for 14 days. (C-D) EDX analysis of M-PAA-Sr hydrogel. (E-F) EDX analysis of mineralized M-PAA-Zn hydrogel.


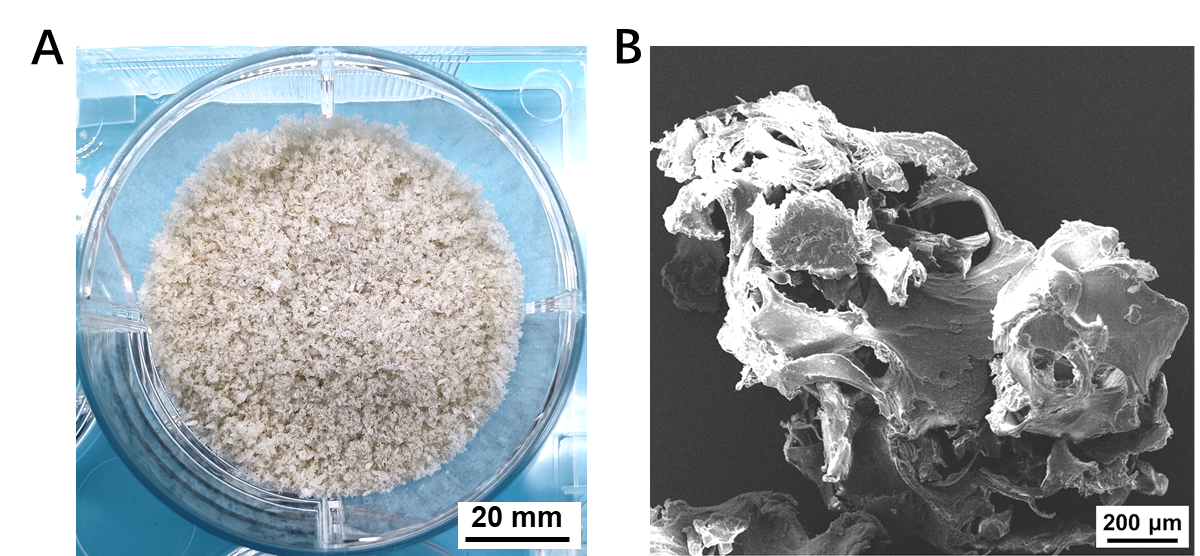


**Fig.S9.** Digital image (A) and SEM image (B) of demineralized bone matrix.


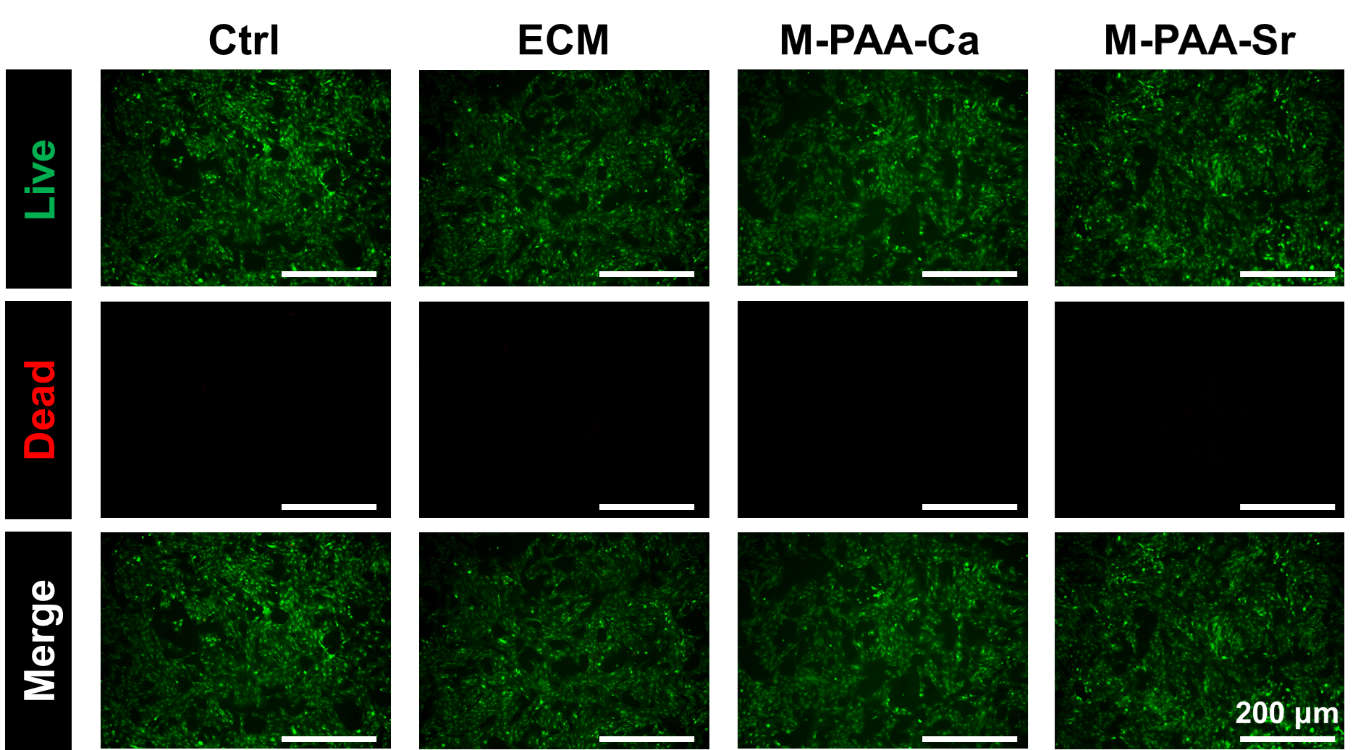


**Fig.S10.**The live-dead staining of BMSCs cultured with the extract of mineralized hydrogel (n=3).


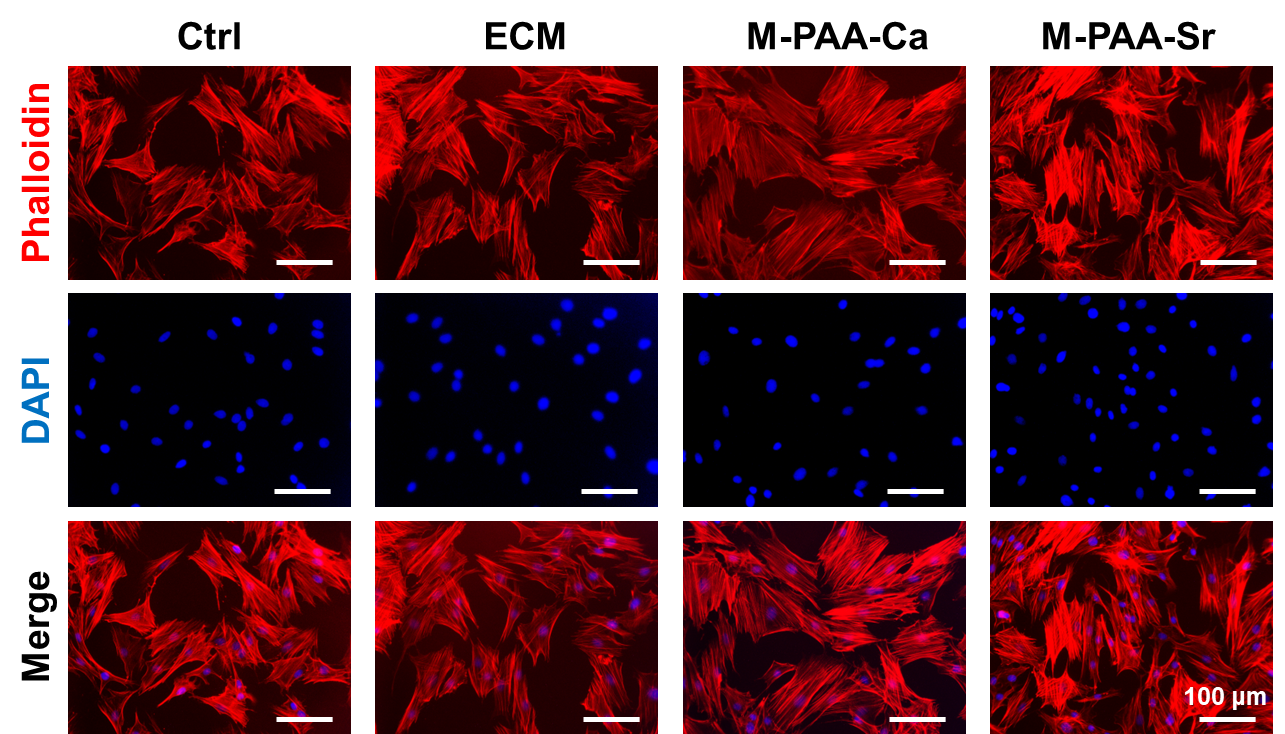


**Fig.S11.** The cytoskeletal staining of BMSCs cultured with the extract of mineralized hydrogel for 3 days (n=3).


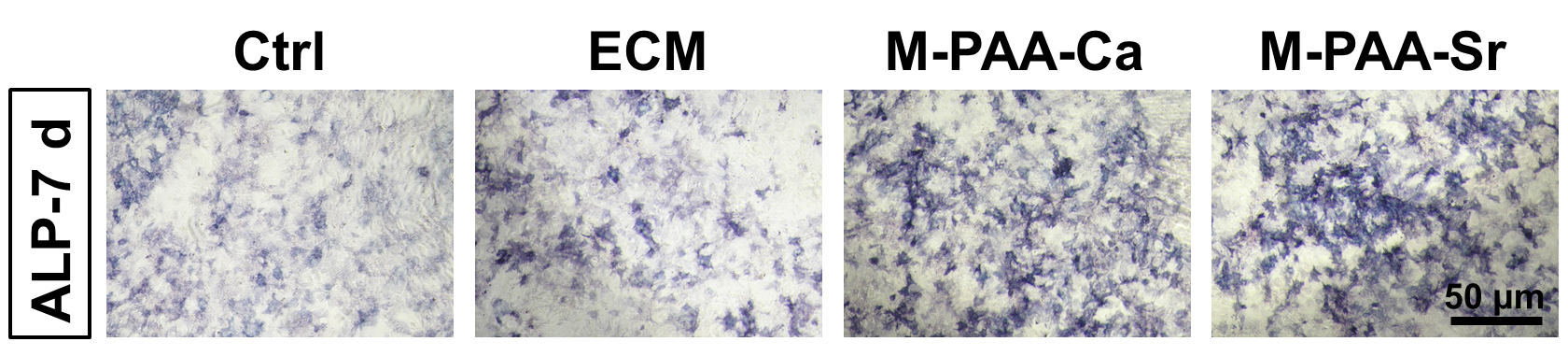


**Fig.S12**.The alkaline phosphatase (ALP) staining of BMSCs cultured with the extract of mineralized hydrogel (n=3).


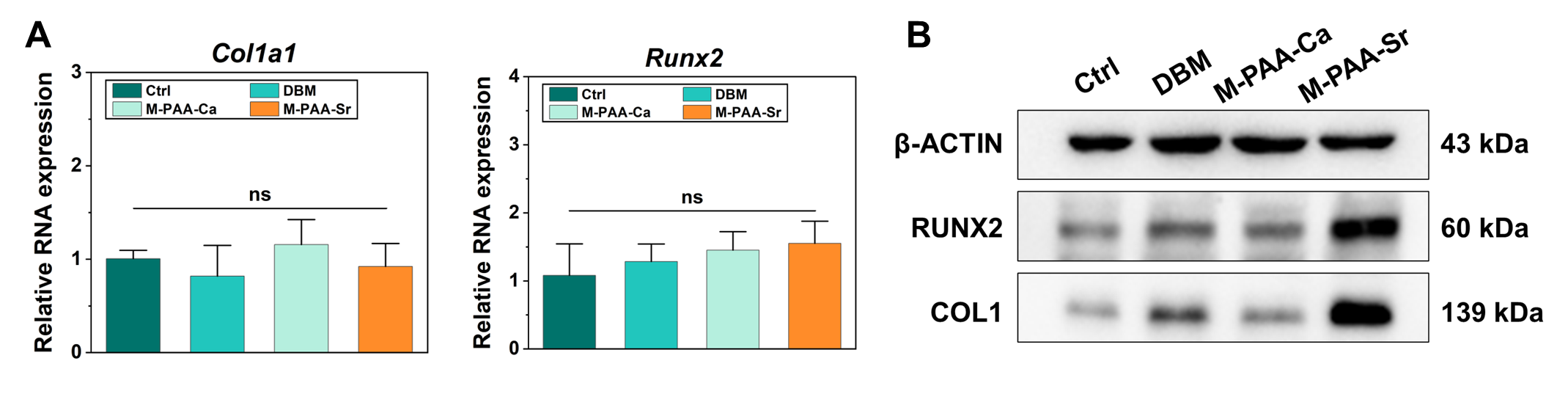


**Fig.S13.** Transcription and translation of factors related to extracellular matrix deposition. (A) Quantification of mRNA levels for Runx2 and Col1a1 in BMSCs by qPCR after culturing on each group for 1 week. ns, no significant difference. (B) Detection of protein levels of Runx2 and Col1 in BMSCs by western blot after culturing on each group for 1 week. Three independent replicates in each group. Statistical analysis was performed using ordinary one-way ANOVA tests with OriginLab, ns= no significance.

**Table S1. Primers used for qPCR of osteogenic genes**

| **Gene Upper primer sequence (5’ to 3’) Lower primer sequence (5’ to 3’)** |
| --- |

*Runx2* GCAGCACGCTATTAAATCCAA GTTGGTGGCATAAAGTATGTG

*Col1a1* CAGGCTGGTGTGATGGGATT CCAAGGTCTCCAGGAACACC

*Gapdh* GACATGCCGCCTGGAGAAAC AGCCCAGGATGCCCTTTAGT

| *Runx2*: runt-related transcription factor 2; *Col1a1*: collagen type I alpha 1 chain. |
| --- |
